# Out-of-Hospital Treatment of Hepatitis C Increases Retention in Care among People Who Inject Drugs and Homeless Persons: An Observational Study

**DOI:** 10.3390/jcm10214955

**Published:** 2021-10-26

**Authors:** Bianca Granozzi, Viola Guardigni, Lorenzo Badia, Elena Rosselli Del Turco, Alberto Zuppiroli, Beatrice Tazza, Pietro Malosso, Stefano Pieralli, Pierluigi Viale, Gabriella Verucchi

**Affiliations:** 1 Infectious Diseases Unit, Department of Medical and Surgical Sciences, University of Bologna, 40139 Bologna, Italy; bianca.granozzi@gmail.com (B.G.); lorenzo.badia@aosp.bo.it (L.B.); erossellidelturco@gmail.com (E.R.D.T.); alberto.zuppiroli@studio.unibo.it (A.Z.); bea.tazza@gmail.com (B.T.); pietro.malosso@gmail.com (P.M.); pierluigi.viale@unibo.it (P.V.); gabriella.verucchi@unibo.it (G.V.); 2 Open Group Society Coop. Soc. Onlus, 40139 Bologna, Italy; pierallis@gmail.com

**Keywords:** PWID, homeless persons, HCV eradication, direct-acting antivirals, out-of-hospital, retention in care

## Abstract

Background. People who inject drugs (PWID) and homeless people represent now a large reservoir of Hepatitis C virus (HCV) infection. However, Hepatis C elimination programs can barely reach these subgroups of patients. We aimed to evaluate and compare the retention in care among these difficult-to-treat patients when managed for HCV in hospital or in an out-of-hospital setting. Methods. In our retrospective study, we categorized the included patients (PWID and homeless persons) into two groups according to whether anti-HCV treatment was offered and provided in a hospital or an out-of-hospital setting. We run logistic regressions to evaluate factors associated with retention in care (defined as the completion of direct antiviral agents (DAAs) therapy). Results. We included 56 patients in our study: 27 were in the out-of-hospital group. Overall, 33 patients completed DAAs therapy. A higher rate of retention in care was observed in the out-of-hospital group rather than in-hospital group (*p* = 0.001). At the univariate analysis, retention in care was associated with the out-of-hospital management (*p* = 0.002) and with a shorter time between the first visit and the scheduled start of DAAs (*p* = 0.003). Conclusions. The choice of treatment models that can better adapt to difficult-to-treat populations, such as an out-of-hospital approach, will be important for achieving the eradication of HCV infection.

## 1. Introduction

The worldwide incidence and prevalence of Hepatitis C virus (HCV) infection has been decreasing since the introduction of the new direct antiviral agents (DAAs) as a form of standard of care [1,2] and the World Health Organization (WHO) has established the global goal of eradicating hepatitis C infection as a public health threat by 2030 [3].

Currently, injection drug use represents the primary route of transmission of HCV infection and the main viral reservoir consists of people who inject drugs (PWID) [4,5], among whom a global anti-HCV seroprevalence of 52.3% has been estimated [6].

Prevalence studies have reported that also homeless persons are at high risk for HCV, mostly as a result of injection drug use [7]. Indeed, PWID tend to experience homelessness or unstable housing with prevalence ranging from 6.7% in Eastern Europe to 50.3% in North America [6].

Homelessness and unstable housing have been recently associated to a greater risk for acquiring infections such as HCV and human immunodeficiency virus (HIV) among PWID when compared to PWID who had stable house [8]. A large meta-analysis has estimated an overall prevalence of HCV infection ranged from 3.9% to 36.2% in homeless people, based on the results of 12 eligible studies [7]. However, there is a scarcity of epidemiological data on the real prevalence of HCV infection in these difficult to treat subgroups [5]. Since HCV elimination programs barely reach these populations, targeted screening programs are necessary to achieve the goal set by the WHO [4]. For a long time, PWID has been regarded as a neglected population due to the concerns about adherence to treatments and poor treatment outcome. Among others, the MISTRAL study has shown how a safe and effective pan-genotypic treatment regimen, particularly with a short duration, could facilitate an increase in accessing treatments for high-risk populations [9,10]. Currently, guidelines for hepatitis C treatment from both the American and the European Association for the Study of Liver Diseases recommends to treat PWID with chronic HCV infection [11,12].

Factors complicating access to care in this population must be addressed including the stigma, the risk for reinfection in PWID, challenges related to incarceration, and housing instability [5,13].

It is also widely recognized that an integrated harm reduction strategy is needed to control HCV transmission and to reduce community viral load [6,14]. By reducing risk behaviors, HCV testing programs that combine screening and counseling can decrease HCV transmission and reinfection after treatment with DAAs [15,16]. The provision of sterile injecting equipment through needle and syringe programs and the enrolment in opioid substitution treatment (OST) are among the primary interventions for reducing HCV reinfection rate among PWID [17].

Recent data have shown that the incidence of HCV reinfections in PWID after achieving sustained viral response (SVR) is low (1.85–22.32/1000 person-years), with higher rates in active drug users [18,19]. 

Screening and confirmation tests, linkage to care, retention in care, prescription of DAAs, and adherence to HCV treatment are priorities for fighting the silent epidemic of chronic HCV infection in PWID and homeless people [9,20]. 

However, PWID and homeless persons have poor access to hospital care due to reduced retention in care and difficulties in accessing traditional screening programs. Therefore, alternative treatment approaches for PWID and homeless people are emerging across Europe [17,20,21,22,23].

In Italy, out-of-hospital care models are emerging with the presence of dedicated doctors, nurses, and peer-educators with experience in drug addiction [24,25,26]. In Italy, the “Stop HCV” project was conceived and conducted in the city of Bologna with the help of the “Open Group-Unità di Strada”, a non-profit organization of harm reduction. The project consisted in offering HCV screening and treatment for hepatitis C using DOT (directly observed therapy), in a population of PWID and homeless people, with this occurring in an out-of-hospital setting. 

The primary aim of our retrospective study was to measure and compare the retention in care rate, (defined as the completion of DAAs therapy) achieved in a group consisting of PWID and/or homeless persons with hepatitis C managed in a traditional hospital setting (i.e., outpatient services) with the retention in care rate achieved in a group of PWID and/or homeless persons but managed in an out-of-hospital setting. 

The secondary aim of the study was to estimate prevalence of patients who started treatment after their linkage-to-care, the time between first visit and the scheduled start of therapy (defined as expected waiting time), and the rate of sustained virological response 12 weeks after the end of treatment (SVR 12).

## 2. Materials and Methods

We carried out a retrospective observational study including patients with HCV chronic infection (i.e., with documented detectable HCV RNA), considered eligible for DAAs treatment, who were active or past intravenous drug users and/or who were experiencing homelessness. In order to test our hypothesis that an out-of-hospital setting might ensure a greater retention in care in difficult-to-treat populations, we compared our outcomes between patients with similar characteristics but treated for HCV in different circumstances (i.e., out-of-hospital and in-hospital services). 

Therefore, we included in the study all the patients with confirmed current HCV infection and history of injection drug use or homelessness who access the out-of-hospital facility where “Senza la C” project was established from January to June 2019. 

This outpatient care model included an initial screening for HCV using saliva rapid tests (OraQuick^®^ Rapid HCV Antibody by OraSure Technologies, Bethlehem, PA, USA) and a pre-test peer counseling offered by educators from Open Group Onlus, the community-based service for harm reduction we mentioned beforehand. Patients also received face-to-face counselling on HCV treatment, prevention, and re-infection risk.

In case of reactive saliva HCV-Ab test, a point of care HCV-RNA test on whole blood (Xpert^®^ HCV VL Fingerstick by Cepheid, Sunnyvale, CA, USA), transient elastography (Fibroscan^®^ by Echosens, Paris, France) and liver ultrasound were performed. Those who resulted HCV-RNA positive were tested through standard blood tests for liver and kidney function and HCV genotype and they were scheduled to start HCV treatment within three to four weeks.

Each of the following visits was conducted at DAAs initiation, after 4 weeks, at the end of therapy, and 12 weeks and 24 weeks after the end of therapy. HCV RNA viremia was performed at each visit in order to rule out any possible relapse or reinfection.

All diagnostic procedures, drug supplying, treatment monitoring, and post-treatment follow-up were conducted in a low-threshold, extra hospital setting by a team of peer educators, medical doctors, and trained nurses. 

We considered as a comparison, a group of patients who met the inclusion criteria and with demographics (age and sex) similar to the group of interest, who had referred to a traditional hospital setting for a visit from May 2017 to August 2018 at our clinic of Infectious Diseases in Bologna (Italy), and were invited by clinicians to start DAAs treatment.

In the out-of-hospital setting, DOT (under the supervision of medical and not-medical staff) was applied, with the support of peer-educators with expertise in management of PWID, in the context of the “Stop HCV” project, which we have already mentioned.

All of the patients included in the study who started anti-HCV treatment, received DAAs for 8 or 12 weeks, according to international guidelines.

We assessed retention in care, defined as the completion of the established DAAs therapy, among our study population. We also measured the expected waiting time, which was defined as the time between the first visit and the scheduled start of therapy with DAAs. With regard to the proportion of population who started and completed treatment for hepatitis C, we observed them for six months after end of treatment. For each subject, we collected the following data at baseline: demographics (age, sex, BMI), stage of liver fibrosis (measured by transient elastography, FibroScan^®^ by Echosens, Paris, France), prior failures to anti-HCV treatment, HCV genotype, HCV RNA viremia, DAAs regimen, data on HIV coinfection when present (i.e., HIV RNA viremia, CD4+T-cells count, current antiretroviral regimen), HBV coinfection (i.e., HBsAg positivity), psychiatric comorbidity, OST, and drug use status (i.e., current PWID or not). INR, bilirubin level, ALT level, creatinine level, and HCV RNA viremia were then evaluated at each scheduled visit.

### Statistical Analysis

Patient characteristics were expressed as median (and Interquartile range, IQR) and percentage when appropriate. The normality of data distribution was assessed with the Shapiro–Wilk test. To compare the characteristics between groups (i.e., in hospital and out-of-hospital setting), we performed the Mann–Whitney U-test and the Chi-squared test (or Fisher Test when appropriate) for continuous and categorical variables, respectively. A *p*-value < 0.05 was considered statistically significant. To evaluate the variables associated with our primary outcome (i.e., retention in care) we performed logistic regression analysis, including in the multivariable model variables which presented a *p*-value ≤ 0.1 at univariate analysis. All of the analyses were performed by using IBM SPSS Statistics for (Windows, Version 24.0, Armonk, NY, USA).

## 3. Results

### 3.1. Patient Characteristics at Baseline

**Figure 1:**
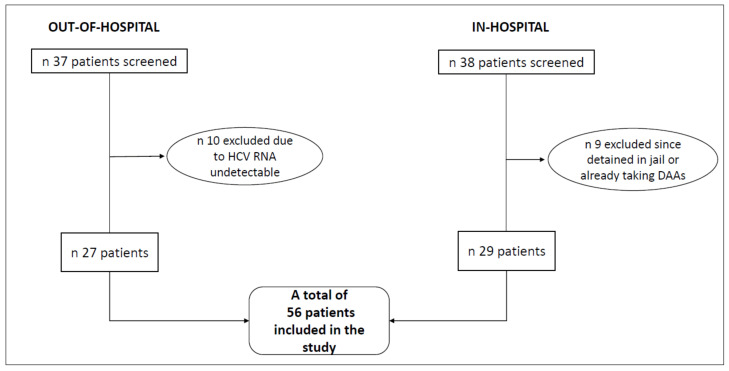
Flow chart of study enrollment.

We enrolled 56 patients who met the inclusion criteria: this included 29 subjects in the in-hospital group and 27 subjects in the out-of-hospital group (as shown in Figure 1). The baseline characteristics are shown in Table 1. The median age was 44.5 years and 92.9% of patients were male. All the subjects in the in-hospital group actively used drugs at enrollment, while only 44.4% of those in the out-of-hospital were PWID (*p* < 0.001). Eleven out of 27 patients referring to out-of-hospital service were experiencing homelessness, whereas only one patient (a 51 years-old female) within the in-hospital setting was homeless, at the time of study participation. All of the patients included in this study had a positive history of intravenous drug use (current or previus).

An overall of 71.4% of individuals (40/56) used OST, with a lower percentage in the out-of-hospital setting rather than the comparison setting (*p* = 0.003). Psychiatric comorbidity was found in 26.8% (15/56) of patients; 58.8% (30/56) of subjects were infected with HCV genotype 1. Five out of fifty-six patients (8.9%) had F3 fibrosis according to Metavir score, while 15.7% (8/56) had documented liver cirrhosis: two out of these eight subjects with an advanced liver disease had decompensated cirrhosis (B8 Child-Pugh class). There was a statistically significant difference in creatinine values between the two groups, with higher levels among those who were treated in the standard in-hospital setting (*p* = 0.003). Thirteen patients (24,5%) were HCV-HIV coinfected: characteristics of this particular subset of patients are shown in Table 2.

### 3.2. Primary and Secondary Outcomes

**Figure 2:**
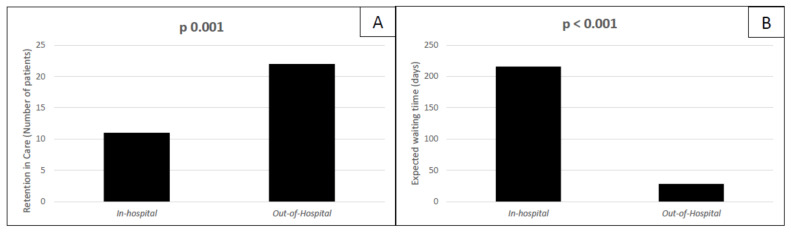
Patients treated with DAAs in our population. Retention in care rates among patients treated for HCV in hospital and out of hospital Panel (**A**); expected days of waiting before DAAs treatment start in the standard in-hospital setting group and in the out-of-hospital setting group panel (**B**).

In our study population, 33 out of 56 patients started therapy with DAAs. The most used HCV regimen was Glecaprevir/Pibrentasvir (73% treated for 8 weeks, 9% for 12 weeks). The remaining patients received therapy with Sofosbuvir/Velpatasvir. All of the 33 patients who started DAAs (corresponding to 60% of the study population) completed treatment with DAAs, with no difference between groups. However, when we analyzed the rate of retention in care (defined as DAAs treatment start and completion, as described in Section 2) among the total study population (56 patients), we observed a higher rate of retention in care in the out-of-hospital group than in standard in-hospital setting (*p* = 0.001), Figure 2A. The expected waiting time was significantly longer in subjects referring to standard in-hospital services (*p* < 0.001), in comparison with the other group (Figure 2B). Among the 33 patients who were treated for Hepatitis C, 93.9% achieved SVR 12 (31/33), with similar SVR12 rates among the two groups (Table 3). The two patients (one in each of the two groups) did not achieve sustained virological response: one experienced a relapse after four weeks from the end of treatment (in-hospital group) and one was diagnosed with HCV reinfection over the follow-up (out-of-hospital group). At the univariate analysis, retention in care was associated only with the out-of-hospital management (*p* = 0.002) and with a shorter expected waiting time (*p* = 0.003), as shown in Table 4. At the multivariate analysis, when we included the covariate “expected waiting time” in the model with “out-of-hospital management” as an exposure variable, the out-of-hospital management did not remain statistically significant as a predictor of retention in care (O.R. 099, *p* = 0.69), while the “expected waiting time” showed a definite trend for association with retention in care, although not still significant (O.R. 0.65, *p* = 0.08). This could potentially suggest that our primary outcome (i.e., retention in care) might be driven by a shorter expected waiting time rather than the setting where patients were managed. When we analyzed the association of parameters with retention in care considering only the 41 patients who were actively using intravenous drugs at time of enrollment, we found that a greater retention in care rate was achieved among those treated out of the hospital (58%) than in the hospital (38%), although not statistically significant (*p* = 0.31). At the univariate analysis, we did not observe any variable associated with our primary outcome, although a shorter waiting time seemed to suggest a higher chance to complete DAAs therapy (Exp (B) 0.995, CI 95% 0.99;1, *p* = 0.055). 

Overall, 37 patients accessed the established out-of-hospital service from January through June 2019 and were all screened for the study. All of them were past or current intravenous drug users or homeless persons. For the comparison group, we considered all the intravenous drug users with detectable HCV RNA who accessed traditional in-hospital service for a visit from May 2017 through August 2018, and we screened a total of 38 patients. 

## 4. Discussion

HCV infection is efficiently spread by injection drug use, and this represents an important public health issue. Furthermore, PWID are very challenging patients to treat due to their difficulties in accessing traditional care in hospital settings and the frequent co-occurrence of alcohol abuse, HIV infection, and psychiatric comorbidities [5,6]. Due to the difficulties in treating PWID, along with often asymptomatic course of HCV infection, there is a risk of underestimating individuals affected by hepatitis C [1]. Similarly, hepatitis C infection represents one of the most prevalent infectious disease among homeless people, and therefore they should be considered a high-risk group and for whom diagnosis and treatment of HCV should be a priority [7]. This lack of data on the real prevalence of HCV infection limits the WHO’s goal of eradicating hepatitis C around the world [2]. Attempts to associate harm reduction interventions simultaneously with the administration of safe and short therapeutic regimens may favor a lowered transmission of the virus and a reduction of liver damage in these populations [9,10]. For these reasons, alternative models of care in out-of-hospital setting are spreading in Europe and Italy, with encouraging results [24,25,26]. Our study showed how an out-of-hospital care model might guarantee a greater percentage of patients starting DAAs with an overall better retention in care for difficult-to-reach groups with HCV infection. In our population, the patients with diagnosis of chronic hepatitis C managed in the out-of-hospital setting were more likely to initiate and complete the therapy, achieving the primary outcome, in comparison to the individuals treated in hospital (*p* = 0.002). Consistently with that, those who were scheduled to start a treatment with DAAs earlier after their first visit were more likely to complete the treatment for HCV infection than those who had to start DAAs with delay (*p* = 0.003). The significantly longer waiting time between the first access to hospital and the scheduled therapy initiation in comparison to the waiting time in out-of-hospital services (216 vs. 28 days) could have represented the major barrier to the “in-hospital” treatment and could explain the lower rate of DAAs treatment in this specific group. All the patients who started therapy were able to complete it (33/33). Therefore, treatment, per se, did not represent an obstacle in completing DAAs therapy in our population. A shorter expected waiting time seemed to increase the retention in care in active PWID (as anticipated). Also, when we focused our analysis on this specific subset of study population (although not statistically significant). We can reasonably assume from our analysis and results that a shorter waiting time is the key for the success of out-of-hospital approach, suggesting that it may play a role as a mediator for a higher proportion of retention in care in the out-of-hospital setting. Moreover, the presence of peer educators may have contributed to improve the linkage to care in the out-of-hospital setting. Starting treatment quickly and in a more individualized way improved the retention in care of PWID [17,20,24,26]. In agreement with our findings, recent research conducted in Vienna on DAAs administration as DOT (given at OST facilities) in PWID showed excellent SVR12 rates (99%) in this difficult-to-treat population, similar to patients with expected high treatment compliance in a standard setting [27]. In our study, although the rate of DAAs therapy completion was lower among patients treated in hospital, when we consider the entire subset of subjects who completed treatment, we observed similarly high virological success rates regardless from treatment setting with no statistically significant differences. The 93.9% of SVR 12 in our overall treated population confirmed the efficacy of regimens with DAAs as reported in the real-world published studies [28]. Small sample size and its retrospective nature are limitations of the study. Moreover, this is a real-world study and we have to acknowledge some baseline differences between the two groups that we compared in the analysis. In particular, all of the patients in the in-hospital group were active intravenous drug users, while less than 50% of the out-of-hospital group was currently using intravenous drugs: for this reason, we ran the same analysis including only active PWID. The presence of educators with expertise in the management of PWID, which are usually lacking in a traditional hospital setting, might also have contributed to the better retention in care achieved in the out-of-hospital facility. In addition, the “Stop HCV project” was interrupted due to a lack of funds. A prolongation of this program would have added relevant data, such as reinfection rate. The results of an effective anti-HCV treatment can be compromised by the risk of reinfection, associated with the persistence of risk behaviors after achieving SVR. For this reason, for a long time, PWID has been regarded as a neglected. However, recent published data have showed how the incidence of HCV reinfection in PWID after the achievement of SVR is low (1.85 to 22.32/1000 person-years) [18,29]. Longer follow-up periods could have certainly provided further data on this population.

In conclusions, our study demonstrated that underserved patients with chronic hepatitis C, historically defined as “difficult-to-treat” groups due to their social instability and risky behaviors, might benefit from new integrated healthcare approaches, such as an out-of-hospital setting where patients may be diagnosed with chronic HCV infection and cured shortly afterwards. The choice of treatment models that can better adapt to difficult populations, such as PWID and homeless people, will be important for achieving the WHO’s goal and therefore further studies are needed.

## Figures and Tables

**Table 1:** Baseline patients’ characteristics, represented for total population and sorted by in-hospital and out-of-hospital setting where chronic hepatitis C was managed.

Characteristics	Total Population (*n* = 56)	In-Hospital Group (*n* = 29)	Out-of-Hospital Group (*n* = 27)	*p* Value
Age (year), median (IQR)	44.5 (35.5–51)	45 (36.5–50.5)	41 (35.0–51)	0.941
Male, *n* (%)	52.0 (92.9%)	27 (93.1%)	25 (92.6%)	1.000
BMI, median (IQR)	22.8 (20.8–24.8)	23.2 (21.0–27.2)	22.6 (20.1–24.5)	0.154
Active PWID	41 (73.2%)	29 (100%)	12 (44.4%)	<0.001
Previous PWID	15 (26.8%)	0 (0%)	15 (55.6%)	0.001
Homeless	12 (21.4%)	1 (3.4%)	11 (40.7%)	0.001
OST, *n* (%)	40.0 (71.4%)	26.0 (89.7%)	14.0 (51.9%)	0.003
Psychiatric comorbidity, *n* (%)	15.0 (26.8%)	6.0 (20.7%)	9.0 (33.3%)	0.370
HBsAg positive, *n* (%)	1.0 (1.9%)	1 (3.4%)	0.0 (0.0%)	1.000
HIV coinfection, *n* (%)	13.0 (24.5%)	10.0 (34.5%)	3.0 (12.5%)	0.108
Liver Stiffness ^1^, kPa, median (IQR)	6.5 (5.1–8.2)	6.8 (5.1–8.6)	6.35 (5.0–8.1)	0.434
Child-Pugh class ^2^, *n* (%)				1
A	6	3	3	
B	2	1	1	
HCV genotype, *n* (%)				0.754
1	30.0 (58.8%)	14.0 (53.8%)	16.0 (64%)	
3	16.0 (31.4%)	9.0 (34.6%)	7.0 (28%)	
4	5.0 (9.8%)	3.0 (11.5%)	2.0 (8%)	
Prior Peg-IFN/RBV failure, *n* (%)	8.9 (14.8%)	2.0 (7.4%)	6.0 (22.2%)	0.250
HCV RNA, log_10_ IU/mL, median (IQR)	6.1 (5.2–6.3)	6.1 (5.4–6.4)	6.0 (5.0–6.3)	0.741
ALT, IU/L, median (IQR)	45.0 (29.0–110)	44.0 (28.3–110)	55.0 (30.0–110)	0.899
Total bilirubin, mg/dL, median (IQR)	0.6 (0.4–0.8)	0.6 (0.4–0.9)	0.6 (0.4–0.8)	0.381
Creatinine, mg/dL, median (IQR)	0.8 (0.7–0.9)	0.9 (0.8–1)	0.7 (0.6–0.8)	0.003
Platelets, ×10^9^/L, median (IQR)	218 (177–266)	202 (152–253)	234 (185–273)	0.108

^1^ assessed by transient elastography (FibroScan^®^), ^2^ variable described only for those patients with documented diagnosis of liver cirrhosis (*n* = 8). Abbreviations: BMI, body mass index; PWID, people who inject drugs; OST, opioid substitute therapy; IFN, interferon; RBV, ribavirin.

**Table 2:** Patients with HIV/HCV coinfection.

Parameters	Total Population (*n* = 13)	In-Hospital Group (*n* = 10)	Out-of-Hospital Group (*n* = 3)	*p* Value
Undetectable HIV RNA, *n* (%)	9 (75%)	8 (88.9%)	1 (33.3%)	0.127
CD4+ cell count/mm^3^, median (IQR)	632 (419–849)	575 (377–891)	688 (545–746)	1.000
ART regimen, *n* (%)				0.931
2NRTI + NNRTI	4 (33.3%)	3 (33.3%)	1 (33.3%)	
2NRTI + INSTI	3 (25%)	2 (22.2%)	1 (33. 3%)	
2NRTI + PI	1 (8.3%)	1 (11.1%)	none	
Others	4 (33.3%)	3 (33.3%)	1 (33.3%)	

Abbreviation: ART, antiretroviral therapy; NRTI, nucleoside reverse transcriptase inhibitors; NNRTI, non- nucleoside reverse transcriptase inhibitors; INSTI, integrase strand transfer inhibitors; PI, protease inhibitors.

**Table 3:** Comparison of primary and secondary outcomes between in-hospital and out-of-hospital settings.

Outcomes	Total Population (*n* = 56)	In-Hospital Group (*n* = 29)	Out-of-Hospital Group (*n* = 27)	*p* Value
Retention in care ^1^, *n* (%)	33 (58.9%)	11 (37.9%)	22 (81.5%)	0.001
Expected waiting time ^2^, days, median (IQR)	42 (28.0–215.3)	216 (168.5–314.8)	28.0 (21.0–28.0)	<0.001
	Treated population (*n* = 33)	In-hospital group (*n* = 11)	Out-of-hospital group (*n* = 22)	
SVR12, *n* (%)	31 (93.9%)	10 (90.9%)	21 (94.5%)	0.6

^1^ completion of DAAs treatment; ^2^ time between the first medical visit and the scheduled DAAs treatment initiation. Abbreviation: SVR12, sustained virological response 12 weeks after end of treatment.

**Table 4:** Univariate analysis of factors associated to the retention in care.

Variables	Univariate Analysis		
	Exp (B)	95% CI	*p*-Value
Age	1.042	0.989; 1.099	0.123
Male sex	0.68	0.088; 5.19	0.71
Metavir F4	0.281	0.031; 2.552	0.26
BMI	0.91	0.79; 1.047	0.18
Homelessness	1.032	0.28; 3.77	0.96
OST	2.71	0.747; 9.87	0.129
Psychiatric comorbidity	0.64	0.19; 2.2	0.48
HIV coinfection	1.43	0.40; 5.1	0.58
Prior Peg-IFN/RBV failure	3.13	0.66; 14.8	0.15
ALT	1.002	0.99; 1.01	0.71
Bilirubin	1.25	0.453; 3.46	0.67
Creatinine	0.128	0.006; 2.77	0.19
Platelets	0.997	0.99; 1.004	0.434
Expected waiting time ^1^, days	0.992	0.987; 0.997	0.003
Out-of-hospital management	0.139	0.041; 0.474	0.002

^1^ time between the first medical visit and the scheduled DAAs treatment initiation. Abbreviations: BMI, body mass index; OST, opioid substitute therapy; IFN, interferon; RBV, ribavirin.

## Data Availability

The data that support the findings of this study are available from the corresponding author, (VG), upon reasonable request.
